# Clinical state tracking in serious mental illness through computational analysis of speech

**DOI:** 10.1371/journal.pone.0225695

**Published:** 2020-01-15

**Authors:** Armen C. Arevian, Daniel Bone, Nikolaos Malandrakis, Victor R. Martinez, Kenneth B. Wells, David J. Miklowitz, Shrikanth Narayanan

**Affiliations:** 1 Jane and Terry Semel Institute for Neuroscience and Human Behavior, University of California Los Angeles, Los Angeles, CA, United States of America; 2 Signal Analysis and Interpretation Lab, University of Southern California, Los Angeles, CA, United States of America; 3 RAND Corporation, Santa Monica, CA, United States of America; Universita degli Studi di Pisa, ITALY

## Abstract

Individuals with serious mental illness experience changes in their clinical states over time that are difficult to assess and that result in increased disease burden and care utilization. It is not known if features derived from speech can serve as a transdiagnostic marker of these clinical states. This study evaluates the feasibility of collecting speech samples from people with serious mental illness and explores the potential utility for tracking changes in clinical state over time. Patients (n = 47) were recruited from a community-based mental health clinic with diagnoses of bipolar disorder, major depressive disorder, schizophrenia or schizoaffective disorder. Patients used an interactive voice response system for at least 4 months to provide speech samples. Clinic providers (n = 13) reviewed responses and provided global assessment ratings. We computed features of speech and used machine learning to create models of outcome measures trained using either population data or an individual’s own data over time. The system was feasible to use, recording 1101 phone calls and 117 hours of speech. Most (92%) of the patients agreed that it was easy to use. The individually-trained models demonstrated the highest correlation with provider ratings (rho = 0.78, p<0.001). Population-level models demonstrated statistically significant correlations with provider global assessment ratings (rho = 0.44, p<0.001), future provider ratings (rho = 0.33, p<0.05), BASIS-24 summary score, depression sub score, and self-harm sub score (rho = 0.25,0.25, and 0.28 respectively; p<0.05), and the SF-12 mental health sub score (rho = 0.25, p<0.05), but not with other BASIS-24 or SF-12 sub scores. This study brings together longitudinal collection of objective behavioral markers along with a transdiagnostic, personalized approach for tracking of mental health clinical state in a community-based clinical setting.

## Introduction

Serious mental illnesses (SMI) such as schizophrenia, bipolar disorder and major depression affect nearly 10 million people in the United States [1] and result in significant symptom burden, lower life expectancy [2], and cost to the healthcare system [3]. These illnesses are challenging to treat, in part due to our limited understanding of underlying biological mechanisms and genetic risk factors [4, 5] as well as their unpredictable relapsing/remitting course [6]. Artificial intelligence methods are increasingly being explored in mental health contexts [7]. In addition, experimental medicine approaches that identify biologically relevant targets (e.g. working memory, speech production, visual perception) as opposed to symptoms have been proposed to help facilitate the translation of basic research findings into interventions [8].

A considerable barrier in achieving the aims of experimental medicine has been identifying objective markers of disease state and traits, including behavioral-based phenotypes [9], that may support transdiagnostic dimensional approaches to disease classification [8]. Trait markers represent properties of the biological system that increase risk for the development of a clinical disorder whereas state markers represent real-time clinical manifestations that may change over time in concert with changes in symptomatic states [9, 10]. Current approaches to measurement of clinical state often rely on the use of question-based scales related to specific symptom domains and functional status. These measures may be subject to recall bias and often require specialized training. They are also often validated at the population-level, making early detection of acute decompensation and prevention of acute care utilization at the individual-level challenging. Increasing the resolution of individual, longitudinal clinical trajectories may support more proactive clinical care and inform our understanding of the biological processes that drive these temporal patterns [10–13].

Novel data sources from mobile devices and sensors, are increasingly being explored to fill this gap [14]. Recently, there has also been growing interest in the use of language as a biologically-relevant, dimensional phenotype [8, 15, 16]. Language provides contextual information relating to an individual’s life experiences and is sensitive to underlying neuro-psychiatric states. For example, increased concrete word use in delirious states and reduced word fluency after sleep disruption has been observed [17, 18]. In the mental health domain, use of specific words including negative or positive emotion and first-person singular words have been associated with depressive states and exposure to traumatic events [19, 20]. Acoustic and other paralinguistic [21] aspects of voice have been associated with depressive symptoms and response to treatment [22–25]. They have also been used to classify manic and depressive states of individuals with bipolar disorder [24, 26, 27], affective states [28], and suicide risk [25]. Behavioral signal processing techniques assess empathy by therapists through analysis of their word choices [29]. In addition, detailed quantification of behavior, including through speech, may be particularly relevant for the measurement of novel features such as complexity [30]. In a number of biological [31] and ecological [32] systems, increased complexity is associated with healthier states. For example, decreased heart rate variability is associated with depression and increased risk for acute cardiac events [33]. While mobile assessment approaches hold promise for improving these kinds of longitudinal assessments, a key to feasibility in real-world clinical settings is sustained engagement which continues to be a challenge [34].

In the current pilot study, we longitudinally collected 4–14 months of speech samples in an outpatient, community-based clinical setting from adults with serious mental illness (primarily mood and psychotic disorders). This was done by creating a novel mobile intervention called MyCoachConnect (MCC), consisting of both interactive voice and web applications. We hypothesized that this assessment method would be feasible and that computed features from patient speech samples would have utility as objective markers of clinical state as measured by a provider-rated global assessment score as well as symptom and functional status self-reported measures. While the feature space for language is broad, we focus primarily on the domains of affective words, complexity and acoustic properties of voice given their prior connection to mental health symptoms [19, 20, 22–24, 26, 27, 35]. Using these features, we tracked the temporal patterns of clinical state and implement personalized models for outcome assessment.

## Methods

### Participants and setting

Participants were recruited from a community-based mental health clinic for adults (age > = 18) with serious mental illness (SMI) and with dual eligibility for Medicare and Medicaid services [36]. Diagnoses were obtained through review of the patient’s medical record. Two types of participants were recruited: providers and patients. A total of 47 patients were enrolled in two phases of this pilot study: an initial open-ended phase (n = 6 patients, n = 3 providers) lasting 18 months (mean engagement length 12 months), and the main pilot phase (n = 41 participants, n = 10 providers). Providers in this study were case managers that provided care to patients as part of the clinic. Case manager roles included keeping in close contact with patients (often interacting at least weekly), providing supportive functions including crisis management and evaluation, and care coordination with their psychiatrist and other physicians. Providers identified potential participants who were then consented and provided instructions on how to access the automated telephone system by study staff over the phone. To recruit a diverse mix of patients we included anyone enrolled in the mental health program who spoke English and had access to at least a public telephone (no personal mobile phone or computer required). Data were collected from 2013 to 2015. Written informed consent was obtained from all participants. All procedures were approved by the RAND Corporation Institutional Review Board (Approval ID: 2012-0703).

### MyCoachConnect telephone tool and mobile app

**Fig 1:**
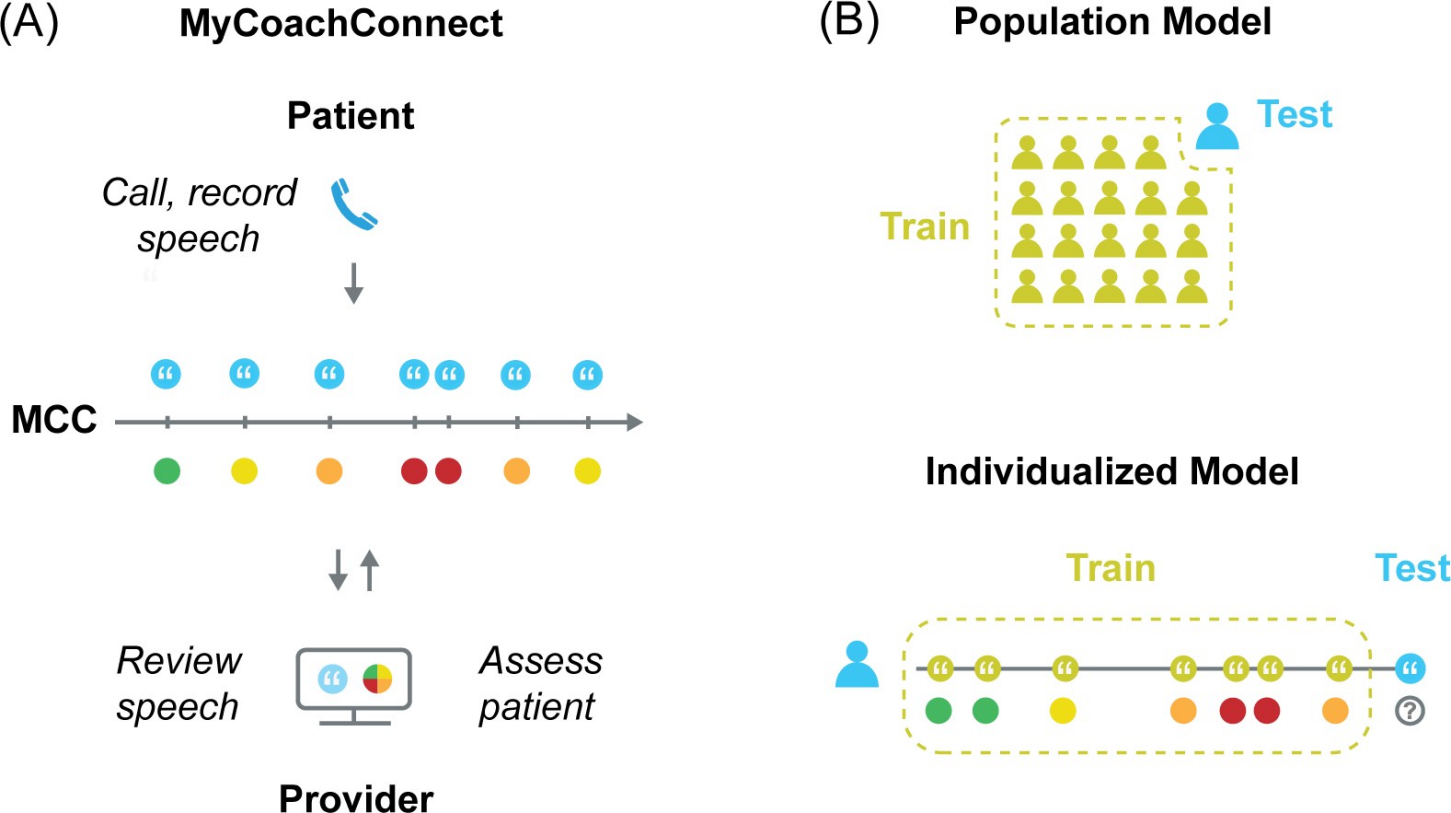
Overview of longitudinal assessment and modeling methods. (A) The MyCoachConnect (MCC) system used to collect speech samples from patients calling into an interactive voice response application. Their providers then used a web application to review speech samples and submit global assessment ratings for each call (B) Comparison of two training methods used. The population-based machine learning model was trained using data from all participants in the study, excluding the test participant. Individualized machine learning model trained on participant’s own data, excluding the test speech sample.

To facilitate more frequent communication from patients and assessments by providers, we created the MyCoachConnect (MCC) system (Fig 1A). This included an interactive voice response app (IVR) used by patients who accessed it by dialing a toll-free number from any phone and authenticating using their personal ID and pin code. Patients were asked to call 1–2 times per week to provide self-ratings and open-ended voice response samples. For each call to the MCC system, patients provided free response answers to three open-ended questions: 1) “How have you been over the past few days?”; 2) “What’s been troubling or challenging over the past few days?”; and 3) “What’s been particular good or positive?”. Patients were asked to speak for 2–3 minutes for each question. They were told that they would be reviewed by study staff and their provider. Patients decided what time of day and day of the week to call based on their preference. Audio recordings for each response were saved and transcribed by study staff using the MCC mobile app. Providers used the MCC mobile app to then review audio recordings and transcriptions of patient responses and complete a single-item global assessment of the patient (see Clinical State Measures below). After completing the study, patients in the main pilot phase completed an exit-survey about their experience using the tool (n = 24/41, 59% completion rate). This included an open-ended feedback question (“What are your reactions about using this tool? What do you think is good or bad about this approach?”). MCC was initially run on a Raspberry Pi server (The Raspberry Pi Foundation, Cambridge, UK) and later migrated to the Chorus mobile application platform hosted at UCLA [37].

### Clinical state measures

The primary measure to assess clinical state was a single-item provider global assessment rating. This measure was completed by the participant’s provider at the clinical site who knew the patient, delivered services, and had access to their medical information. Providers used the MCC mobile app to provide a single, global assessment rating at the time of each patient’s call (“Overall, as this patient's provider, how do you think this patient was doing around the time of the call given everything you know about the patient? (1 is the worst and 10 is the best)”). This measure, similar to the Global Assessment of Functioning (GAF) scale from the DSM-IV, aims to provide a brief rating of overall health status and does not distinguish between symptom severity and health-related functioning [38]. However, we chose to use this single item as our primary clinical state measure as it was feasible for providers in clinical settings to rate frequently and could be used for participants across SMI conditions. We also used as secondary measures the BASIS-24 and SF-12 administered over the phone by study staff in the main pilot [39]. The BASIS-24 is a broad psychiatric symptom scale including subdomains for depression, interpersonal problems, self-harm, emotional lability, psychosis and substance use. To measure health-related functioning and well-being, we utilized the SF-12, a global health functioning and well-being scale with both mental health and physical health subdomains (S1 Data).

### Lexical and acoustic features

We computed features from patient speech samples to capture lexical content, lexical complexity, and vocal expression using methods which show competitive performance across several speech assessment tasks [40, 41]. As the potential feature space is very broad, we selected specific sets of features informed by clinical considerations (e.g. using features related to mood and health status) and prior studies. All features were selected a priori without preliminary analysis to guide feature selection. To quantify language content, we used two overlapping methods. First, we extracted lexical norms, which quantify language content relative to expectations, including arousal, valence, positivity, negativity, objectivity, concreteness, age of acquisition, pronounceability, and gender ladenness. Second, we selected features from the Linguistic Inquiry and Word Count (LIWC) [42] toolbox which included features related to affect, religiousness and health-related words. As the degree of complexity has been associated with the health of biological systems [31] we extracted several features related to complexity. We measured lexical complexity using conventional readability measures (reading ease and grade level) [43], readability indices [44–46] including the Subjective Measure of Gobligook (SMOG) index which is widely used in health literacy assessment [47], and the number of difficult words (defined as having more than six syllables). We also used latent semantic analysis (LSA) to generate features representing semantic coherence, a measure of similarity between nearby verbal phrases. LSA uses patterns of words contained in adjacent phrases as a measure of semantic structure to analyze coherence between these phrases. LSA coherence features have previously been associated with clinical ratings in schizophrenia [48] and bipolar disorder [24]. Motivated by prior reports [49], nine acoustic features of voice were computed using Praat [50] and custom analysis scripts. Acoustic features include measures of pitch, intonation, vocal formants, fundamental frequency, and inter-word pause length. Audio samples were rejected if human transcribers could not understand the majority of the content. In total, we computed 210 features (70 features applied to each of the three voice responses per call) (S1 Table). Correlational feature analysis is commonly used in machine learning research to provide insights into the features that the model may be utilizing in making decisions. We compute the pairwise Spearman’s correlation at the population-level between the 210 features and the provider ratings for all samples (n = 1101). Given the large number of features, we rely on a Bonferroni correction. Similarity in feature utility is examined across subjects by computing the pairwise correlations of feature values with provider ratings for each individual. While there were three audio prompts per call, features from each prompt were combined to generate the full set of 210 features per call, which were used in the model to compute the clinical rating for that call. Therefore, each call and its related features were considered independent of one another.

Additionally, to quantify the similarity within individuals of the degree each feature was correlated with provider ratings, we utilized a two-tailed, William’s tests comparing feature values from the first half to the second half of samples collected within individuals over time.

### Clinical state tracking

Support vector machine (SVM) methods were used to create the machine learning models from the total set of 210 computed features per patient call to assess the three measures of clinical state including the global provider assessment ratings, symptom severity (BASIS-24), and functional status (SF-12). Support vector regression was performed using the L-2 regularized optimization from Liblinear [51]. Parameters were tuned using a 2-layer cross-validation approach operating within the training fold. A grid search was utilized to optimize the cost (c in {0.01,0.1,1,10}) and epsilon ({p in {0.01, 0.1, 1}).

**Fig 2:**
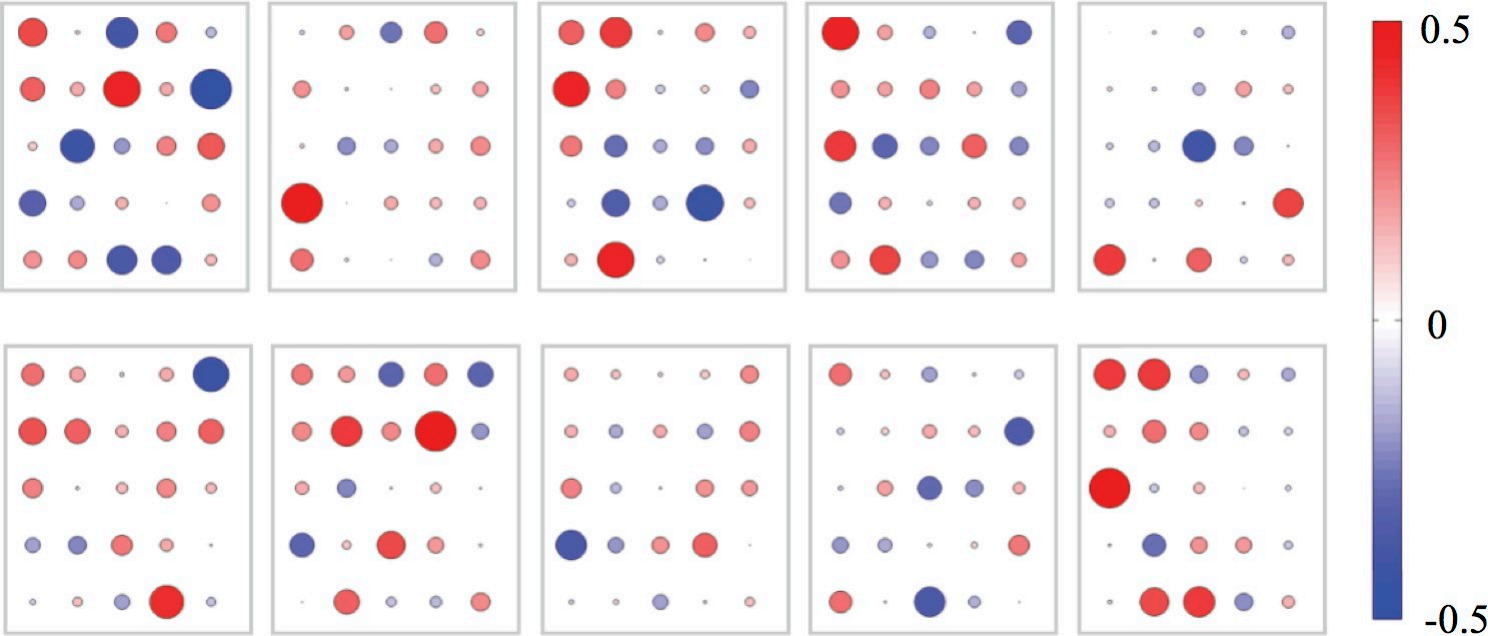
Patient-specific correlation patterns for speech features. Correlation patterns between speech features and provider global assessment ratings for the top 25 features with the highest average correlation at the population level.

As the primary aim of the study was to track changes in clinical state over time, we used the global provider assessment ratings as our primary outcome, which was assessed for each participant call. We compared two different approaches to training the model. First, we implemented population-based training using samples from all participants except the test participant (leave-one-subject-out cross-validation). This ensures independence between the training data and the test data. Second, we implemented an individually-trained approach using data from an individual participant only (leave-one-sample-out cross-validation) (Fig 1B). With the individually trained model, samples from an individual are used to train the model except the sample used to test the model. Because we are using cross-validation, there is no concern of inflated performance (e.g., as with inflated R^2 seen with adding additional features to an ordinary least squares model without cross-validation). With cross-validation, having more features does not guarantee higher performance. For provider global assessment ratings, we tested two types of assessment: 1) concurrent–using the speech sample to determine the provider rating during the same set of calls, and 2) forecasting–using speech samples to determine subsequent provider ratings (Fig 2C).

For modeling of the BASIS-24 and SF-12 scores, we trained a population-based model due to the limited number of surveys obtained. Since the assessments did not directly coincide with call occurrences, we matched any test to the nearest call occurring within two weeks. For the population model (n = 47 participants, n = 1101 calls) scenario, the baseline is to assume no correlation. However, for the individual model, the baseline is the mean of all ratings for an individual apart from the call that is being assessed. For the individual models, we also required 35 voice samples per participant, which resulted in 10 participants used in the model with 514 provider ratings. The threshold of 35 voice samples was chosen based on our preliminary analysis showing that correlation of the model improved with training up to about 35 samples and then plateaued. Given that clinical state is often measured on the timescale of weeks, we primarily created models using the 4-period moving average of both provider ratings and computed features for both the population (n = 39 participants, n = 215 4-period samples) and individually-trained (n = 10 participants, n = 116 4-period samples) models. We used non-overlapping windows for the averaging to ensure sample independence.

Spearman’s correlation coefficient was used to assess the degree of covariance between the models’ computed values of the clinical ratings and actual clinical ratings. We then assessed the degree of covariance of the baseline models and the actual clinical ratings. For the population-based model, correlations were averaged across subjects. For the individually-trained model, correlations were averaged across samples. One-sided statistical tests were used when an improvement in model correlation over baseline was hypothesized.

## Results

### Patient engagement and speech sample characteristics

Demographic and illness characteristics of the patient population (n = 47) are summarized in Table 1. Patients had a diagnosis of bipolar disorder (n = 14, 30%), major depressive disorder (n = 15, 32%), schizophrenia (n = 14, 28%) or schizoaffective disorder (n = 14, 30%). Patients experienced considerable symptom burden (BASIS-24 summary score of 1.5 compared to 0.54 general population norm) [33] and decreased functional status (population MCS-12 and PCS-12 mean scores were approximately 1 SD below the population mean for this age group).

**Table 1:** Sample characteristics.

Participants	Total (n = 47)
Female (%)	21 (45%)
Age (SD)	51.1 (12.5)
**Race, Ethnicity**	
White, Non-Hispanic	24 (51%)
African American	18 (38%)
Hispanic	5 (11%)
**Diagnoses**	**n (%)**
Bipolar disorder	14 (30%)
Schizophrenia	13 (28%)
Schizoaffective	14 (30%)
Major depressive disorder	15 (32%)
*+substance use disorder*	18 (38%)
**Clinical characteristics**	**Mean (SD)**
BASIS-24 (n = 42)	1.6 (0.5)
Depression and Functioning	1.7 (0.8)
Interpersonal Problems	2.6 (1.0)
Self-Harm	0.2 (0.7)
Emotional Lability	1.8 (1.1)
Psychosis	1.2 (1.1)
Substance Use	0.3 (0.3)
MCS-12 (n = 39)	42 (11.5)
PCS-12 (n = 39)	39 (6.4)

In the main pilot phase (n = 41 patients), patients were followed for 16 weeks. There were nine patients who withdrew early from the study including five that were lost to follow- up, three that indicated it took too much time or were not interested in calling and one who was admitted to an inpatient psychiatric hospital. During the 16-week pilot, the average engagement length of participants was 14.7 (S.D. 6.5) weeks, who called an average of 19.7 (S.D = 12.3) times. Patients reported in the exit survey that usability was favorable, with most agreeing or strongly agreeing that they thought it was easy to use the MCC system (n = 23 of 24 respondents, 96%), felt their provider was better able to help them because of using it (18/24, 75%) and would use it in the future even if not compensated for their time (n = 21/24, 88%). Feedback descriptions of participant experiences were also positive with 22 patients reporting positive statements (92%), 1 (4%) reporting a negative statement (“technical issues”) in addition to positive statements, and 2 (8%) did not provide any statements. Representative statements were selected from responses, including reports of loneliness that was partially address through improved sense of connection to their provider: “It surprised me, I got some good out of it”; “It was great. It gave me a chance to vent”; “Not talking to an actual person helped organize thoughts”; “An outlet to talk about your feelings if you're alone or if you don't have anyone to listen to you”; “Helped me let my stress out, took a weight off my back of committing suicide or drinking again. Think it helped because I had someone to talk to.”

We collected a total of 1101 voice samples consisting of 117 hours of free speech audio recordings from 47 patients. The mean global provider rating was 6.1/10 (S.D. = 1.6, skew = -0.34, kurtosis = 0.19). The average number of words per response was of 117.7 (S.D. = 115.9) words and the total words per call (three responses per call) was of 337.1 (S.D. = 302.6) words.

### Correlation analysis of speech features

Correlations between features and provider rating for the top performing features at the population-level are listed in Table 2. Features related to affect were most informative with higher provider ratings associated with language having less negative emotion (-0.36) and more positive emotion (0.34) according to LIWC percentages (and similarly for the affective norms of valence, negativity, and positivity). Higher provider ratings were also associated with more complex word use, for example more difficult word usage (0.21), a higher readability index (0.14), and more variability in LSA-based coherence (0.16). Acoustic features were also correlated to provider rating including the F2 harmonic (0.18) and harmonicity (-0.12). The average absolute value of correlations for all features for the population was 0.23. Interestingly, this correlation increased when averaging over multiple assessments (for example, 0.44 correlation of the 8-period moving average values of provider rating to speech features).

**Table 2:** Acoustic and linguistic feature correlations with provider global assessment.

Feature	Set	Functional	Correlation
Negative emotion words	LIWC	% words	-0.36
Positive emotion words	LIWC	% words	+0.34
Valence	Lexical Norms	Mean	+0.32
Negative	Lexical Norms	Mean	-0.32
Positive	Lexical Norms	Max	+0.26
Difficulty of words	Complexity	Mean	+0.21
Religious words	LIWC	% words	+0.20
Gender ladenness	Lexical Norms	Min	-0.20
Arousal	Lexical Norms	Min	-0.19
2^nd^ vocal formant	Acoustics	Mean	+0.18
Sad words	LIWC	% words	-0.16
Coherence (latent semantic analysis)	Complexity	Stdv.	+0.16
SMOG Index	Complexity	Mean	+0.14
Harmonicity	Acoustics	Median	-0.13
Assent	LIWC	% words	+0.12

LIWC, Linguistic Inquiry of Word Count toolkit; SMOG, Subjective Measure of Gobbligook Index.

While certain features were informative across all subjects (e.g., negative and positive emotion words), there was considerable variation at the individual level in which features were most correlated with the clinical state of that individual. This variability between individuals is shown in Fig 1, where the correlation to provider ratings for the top 25 features at the population-level are displayed for the 10 patients used for the personalized training model, each displaying a unique pattern. To quantify the degree of similarity, we calculated the pairwise cross-correlation of the set of feature correlations to provider ratings. Individuals exhibited an average pairwise correlation with other individuals of only 0.05. Despite this difference *between* individuals, we also observed that *within* individuals, the degree that speech features were correlated with clinical state was largely stable over time. There was no statistically significant change in the correlation between the first half of samples and the second half within an individual for 93% of features (p<0.05).

**Fig 3:**
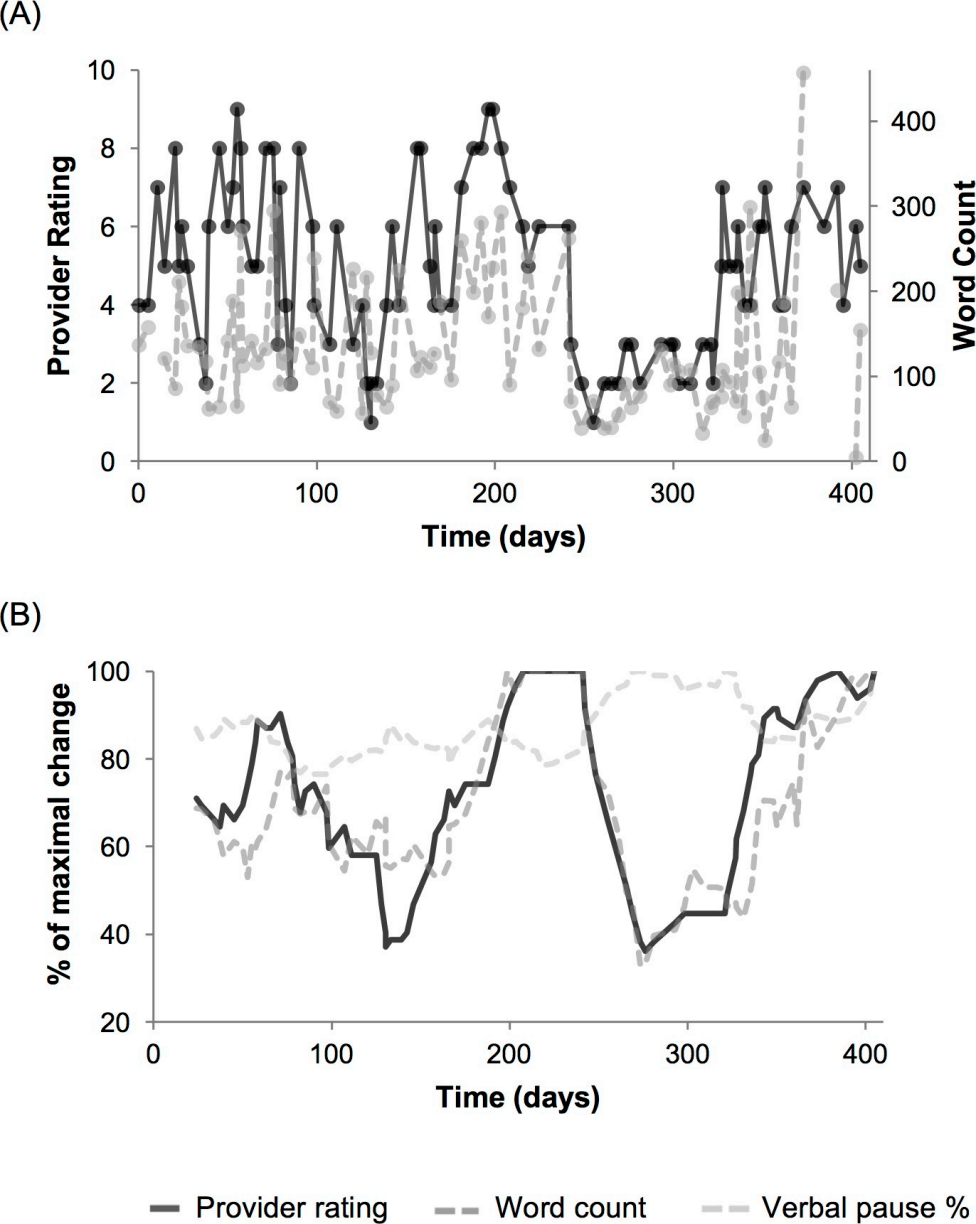
Covariance of speech features and clinical state over time. (A) An example of clinical state (provider global assessment rating, black line) transitions within an individual patient over time compared to the individual’s highest performing linguistic feature (word count per speech sample, dotted grey line) for each call to the MCC system. (B) Increased correlations between clinical state and speech features over time highlighted through percent of maximal change of 8-period moving averages for provider rating (black line), word count (dotted grey line), and verbal pause percent (dotted light grey line) for the same patient and period.

An illustration of critical speech features co-varying with associated provider rating for an individual subject is shown in Fig 3, displaying raw data (Fig 3A) as well as the smoothed, 8-period moving average values of features and provider rating (Fig 3B). In this example, there was a 0.33 correlation between the top performing feature (speech sample word count) for this individual and provider ratings. However, using the 8-period moving average of provider rating and computed features identified both a more consistent temporal trend in provider ratings and an increase in correlation of speech features to provider ratings (r = 0.80).

### Tracking clinical state

Our primary analyses explored machine learning models to track clinical state over time using the computed features from patients’ speech samples. In Table 3, we compare the degree of correlation with clinical state (assessed by the provider global assessment ratings as well as symptom and functional status measures) using models trained on the population as well as personalized models trained within individuals.

**Table 3:** Clinical state tracking of provider global assessment ratings using speech features.

**A. Population model**	**Correlation** ^**a**^ **(P-value** ^**b**^**)**
Concurrent assessment	0.44 (p<0.05)*
Forecasting assessment	0.33 (p<0.05)*
**B. Personalized model**	**Correlation** ^**a**^ **(P-value** ^**b**^**)**
Concurrent assessment	0.78 (p<0.05)*
Forecasting assessment	0.62 (p>0.05)

^a^ Correlation assessed using Spearman’s rank-order coefficient

^b^ p values calculated using 2-tailed t-test compared to baseline models

Number of observations reported as n.

For the population model (n = 47 participants, n = 1101 ratings), we used data from all patients for training the model except the test subject. Computed scores from the model had a correlation with provider ratings of 0.44 (p<0.05). To explore the ability for the model to forecast future clinical states, we used speech samples from one sampling period as the input to the model to compute the subsequent sampling period global assessment rating. The computed results from the model demonstrated statistically significant correlations with actual provider ratings for these future clinical states (rho = 0.33, p<0.05). For the BASIS-24, correlation was statistically significant for the summary score (rho = 0.25, p<0.05), as well as for the depression (rho = 0.25, p<0.05) and self-harm (rho = 0.28, p<0.01) sub scores. We achieved a similar correlation in computing the mental health subscale of the SF-12 (rho = 0.25, p<0.05), but not for the physical health subscale or the other four out of six BASIS-24 sub scores. The model was not able to classify patients according to diagnostic group (32.5% unweighted average recall, p = 0.2).

Given the difference in patterns of correlation between speech features and clinical state between individuals, we then explored within subject analysis using only data from individuals to train the model, including subjects with at least 35 voice samples to ensure sufficient data to train and time to observe within subject variability in clinical state (n = 10 participants, n = 514 ratings). This personalized model demonstrated statistically significant improvement in correlation with provider global assessments than the baseline and population-based models (rho = 0.78, p<0.01). Forecasting future clinical states using the individual model had a correlation of 0.62, although this did not exceed the baseline model correlation (0.66) of the subject’s average assessment ratings.

## Discussion

This study demonstrates that self-reported speech samples are feasible to collect longitudinally in a community-based clinical setting from patients with serious mental illness. It also shows that computed lexical and acoustic features from those samples can be used to track within-individual changes in mental health states over time. Using an individually-trained algorithm, prediction models resulted in a high correlation (up to 0.78) between predicted and actual clinical states, the latter based on providers’ global assessment ratings. Correlation with secondary clinical measures provided mixed support for our hypotheses. Using a population model, we demonstrated statistically significant correlations between the model and actual scores only with the summary, depression and self-harm sub scores of the BASIS-24 as well as the mental health sub score of the SF-12. However, there were no statistically significant correlations between the model and four of the six BASIS-24 sub scores or the physical health sub score of the SF-12. Finally, the population model demonstrated statically significant correlation between computed and future provider global assessment ratings using speech samples from previous calls. These prospective findings hold promise for creating clinical interventions that forecast patient needs and proactively address them.

These results may have important clinical implications. First, computed scores from individually-trained model achieved the highest correlation of 0.78, indicated a considered a strong correlation. This is similar to the interrater reliability of instruments used to measure mental health status in practice [52]. Because the personalized model requires a period of individual-level training to achieve this degree of correlation, it may also inform how care is delivered. For example, health organizations may recommend more frequent assessments for individuals with SMI early in their care to set this baseline and to train their personalized model. This could then be followed by routine in-person assessments paired with remote interactive voice response engagement and monitoring. The population model demonstrated moderate correlation (0.44) with provider ratings. While prediction using the population model could be improved through subsequent studies, it may be useful as a screening approach to identify patients that may have clinical needs and then follow-up with more sensitive measures.

We used the strength of the correlation between computed ratings and clinical outcome ratings as the measure of the model’s ability to assess clinical state. This represents the degree of covariance between computed and actual ratings, allowing us to track relative changes over time, but it is not a direct measure of its accuracy in predicting a specific score. This method is consistent with our goal of piloting an assessment approach that would be feasible in community-based clinical settings and useful for clinical state tracking over time. Additionally, this approach uses dimensional clinical outcome ratings, which are notably different from classification tasks involving discrete states (for example euthymic vs. manic states in bipolar disorder). Using classification of discrete states, one can utilize additional methods to assess the predictive ability of the model (e.g., determining the sensitivity, specificity and area under the curve). With larger datasets, hierarchical linear modeling could be used to explore potential clustering of individuals and to model non-linear changes over discontinuous time periods. This improved temporal modeling could be of particular importance in improving the ability of the model to forecast future clinical states.

Interestingly, despite a high degree of stability in speech features within individuals over time, there was little correlation between individuals regarding which speech features were most correlated with their clinical state. This suggests that the pattern of word choice as it relates to mental illness/wellness may be specific to individuals. The importance of taking individual variability into account was demonstrated by the personalized model’s improved correlation with provider global assessments when utilizing an individual’s own data to learn which features are most related to their clinical states. This suggests that both population-based approaches, along with augmentation from learning at the individual level, may have advantages in informing computational methods that utilize behavioral markers. Future studies may explore if certain features are common across individuals (possibly within diagnostic groups or associated with other dimensional constructs). Of note, complexity of language, reflected in features such as age of acquisition and readability of words, were also correlated with clinical state. Prior studies have shown that complexity is reduced and temporal patterns more deterministic in individuals with mental illness, including bipolar disorder [53], depression [30], and psychosis [54, 55].

Voice samples were collected actively, with patients choosing to call an interactive voice response system rather than passive sensor mechanisms. Because of this, a smartphone was also not required for participation in this study, reducing the technical barriers for participation, which may be particularly important for engagement within community-based clinical settings. However, this also requires more active involvement of the participant. It will be important to explore how these methods generalize to other populations. While this preliminary pilot study had a broad, transdiagnostic population across serious mental illness diagnoses, future studies may investigate these methods and factors that affect adherence with healthy volunteers, those with mild or moderate mental health symptoms, or with chronic physical conditions.

This study only examined a subset of potential acoustic features of speech. Acoustic features have been used in prior studies to classify hypomanic (AUC 0.81) versus depressive (AUC 0.67) states in bipolar disorder [24, 26]. In addition, acoustic pitch variability as well as changes in pause time between words have been shown to be significantly correlated with depression scores [35], and multiple acoustic features have been used to predict response to treatment in depression [23]. The second vocal formant, F2, was observed in our study to be higher for patients with better clinical states, which is consistent with previous findings that the vowel space (comprised of F1 and F2) is higher for less-depressed patients [48]. However, the quality of audio features could also have been reduced from the telephone-grade audio recordings which have less spectral resolution than high quality audio recordings. It is possible that using additional acoustic and other paralinguistic features [23, 25, 56], along with higher quality audio samples, may be more informative. There are also a number of emerging projects that explore mobile sensing approaches in individuals, especially related to bipolar disorder. Examples include the use of textiles with embedded sensors to collect motion and physiological data [57], typing dynamics from mobile phone keyboard use to predict affective states [58], and acoustic features from passively collected voice samples [59]. An exciting future direction will be to work towards integrative computational models including a more complete set of linguistic, paralinguistic, behavioral, sensing, and neural features.

There were several challenges highlighted in the assessment of individuals in clinical settings. First, our primary clinical outcome measure was a global assessment rating as it provided a practical method to quickly assess global clinical state given time constraints of clinical staff. While it is adapted from the widely used Global Assessment of Functioning scale used in DSM-IV, it was not previously evaluated for measure constructs such as validity and reliability [37]. Because the rating was based on a clinician’s assessment, there could be variability in the rating, similar to that observed with the GAF scale [52]. However, we chose it because it is commonly used in community care settings and enables assessment across multiple diagnoses, important factors for this study. It also represents a broad integration of symptoms and functional status. While this aligned with the goal of the study to assess the global clinical state of an individual, this single-item measure is not able to differentiate between specific psychiatric symptoms such as depression, mania or psychosis. This is a general challenge in psychiatry where measuring the “ground truth” of true clinical state can be difficult [60]. However, this global measure is consistent with our transdiagnostic approach as it does not rely on disease specific conditions. There are also precedents in other settings for using single-item, global measures of health status including the overall health status question from the SF-12 that has shown validity across physical and mental health conditions as a single-item measure [61]. The distribution of our provider ratings was not evenly distributed across all possible scores. This skew should not affect our results since we use a correlation-based metric. Future studies can analyze whether this distribution is an accurate representation of the target test population. If needed, any imbalance can be addressed through oversampling of patients in relevant parts of the scale.

This study did not have the participant sample size to explore the variability in model performance between participant characteristics such as specific diagnostic categories, native language spoken, educational level, and race. In addition, we cannot determine the strengths or weaknesses of the features and algorithms with regards to symptom-specific states (e.g. euthymic vs. depressed states) or differences compared to healthy volunteers. Future studies may also explore additional modeling approaches such as the use of neural networks and recent word embedding techniques [28, 62, 63]. Longer observation periods would enable capturing acute clinical events (such as hospitalizations) and additional clinical state transitions within individuals (e.g. from stable to decompensated states).

The predictive models were also not able to significantly predict the physical health subscale of the SF-12 or the subscales of the BASIS-24 other than the depression and self-harm subscales. This may be related to the use of mostly affective features that would relate more to affective states (and therefore the BASIS-24 sub scales of depression and self-harm). It may also be due to the general symptom measure used (BASIS-24), and correlation may improve with symptom-specific measures. The performance may also be related to the relatively limited number of assessments that were able to be collected in this study for the BASIS-24 and SF-12 as compared to the primary provider global rating, which was collected for each phone call. However, we cannot rule out the possibility that this approach may be more effective for depressive states than other mental health states.

This study brings together longitudinal collection of objective behavioral features derived from speech (lexical and acoustic) along with a global, transdiagnostic and personalized approach to prediction of clinical state in a community-based clinical setting. Language encodes a rich and contextual feature space that holds promise as an objective behavioral marker of clinical outcomes across mental health diagnoses at both the population and individual level. This study supports the importance of considering approaches that pair objective behavioral data with clinical care to support personalized, and pragmatic approaches to improving care and our understanding of an individual’s clinical state. Ultimately, these objective markers may support translational efforts to better understand the underlying genetic, neurobiological and external factors driving changes in these states.

## Supporting information

S1 Data Data for BASIS-24 and SF-12 baseline patient measures. (XLSX)

S1 Table Computed speech features. List of all computed speech features including category of feature and unit of measure.
(DOCX)
