# Supplementary material for: Clinical state tracking in serious mental illness through computational analysis of speech
Source: PLoS One. 2020 Jan 15;15(1):e0225695. doi: 10.1371/journal.pone.0225695 (PMC6961853; doi:10.1371/journal.pone.0225695)
Supplement: S1 Table — List of all computed speech features including category of feature and unit of measure. (DOCX) [file pone.0225695.s002.docx]

**S1 Table. Summary of computed lexical and acoustic features**

| **Feature Set** | **Feature** | **Functional** |
| --- | --- | --- |
| SentiWordnet | Negativity | Average |
| SentiWordnet | Negativity | Max |
| SentiWordnet | Negativity | Min |
| SentiWordnet | Negativity | Stdv. |
| SentiWordnet | Objectiveness | Average |
| SentiWordnet | Objectiveness | Max |
| SentiWordnet | Objectiveness | Min |
| SentiWordnet | Objectiveness | Stdv. |
| SentiWordnet | Positivity | Average |
| SentiWordnet | Positivity | Max |
| SentiWordnet | Positivity | Min |
| SentiWordnet | Positivitiy | Stdv. |
| LIWC | Assent | % Words |
| LIWC | Health | % words |
| LIWC | First person singular | % words |
| LIWC | Negative emotion | % words |
| LIWC | Positive emotion | % words |
| LIWC | Religiousness | % words |
| LIWC | Sadness | % words |
| LIWC | Word count | Count |
| Lexical Norms | Age of Acquisition | Average |
| Lexical Norms | Age of Acquisition | Max |
| Lexical Norms | Age of Acquisition | Min |
| Lexical Norms | Age of Acquisition | Stdv. |
| Lexical Norms | Arousal | Average |
| Lexical Norms | Arousal | Max |
| Lexical Norms | Arousal | Min |
| Lexical Norms | Arousal | Stdv. |
| Lexical Norms | Concreteness | Average |
| Lexical Norms | Concreteness | Max |
| Lexical Norms | Concreteness | Min |
| Lexical Norms | Concreteness | Stdv. |
| Lexical Norms | Gender Ladeness | Average |
| Lexical Norms | Gender Ladeness | Max |
| Lexical Norms | Gender Ladeness | Min |
| Lexical Norms | Gender Ladeness | Stdv. |
| Lexical Norms | Pronounceability | Average |
| Lexical Norms | Pronounceability | Max |
| Lexical Norms | Pronounceability | Min |
| Lexical Norms | Pronounceability | Stdv. |
| Lexical Norms | Valence | Average |
| Lexical Norms | Valence | Max |
| Lexical Norms | Valence | Min |
| Lexical Norms | Valence | Stdv. |
| Counts | Number of sentences | Count |
| Counts | Number of tokens | Count |
| Counts | Words per sentence | Median |
| Complexity | Readability | n/a |
| Complexity | LSA Coherence | Average |
| Complexity | LSA Coherence | Max |
| Complexity | LSA Coherence | Min |
| Complexity | LSA Coherence | Stdv. |
| Complexity | LSA Coherence | Average |
| Complexity | LSA Coherence | Max |
| Complexity | LSA Coherence | Min |
| Complexity | LSA Coherence | Stdv. |
| Complexity | Difficult words | Count |
| Complexity | Flesh-Kincaid Reading Level | n/a |
| Complexity | Flesh-Kincaid Reading Ease | n/a |
| Complexity | Gunning fog index | n/a |
| Complexity | SMOG Grade | n/a |
| Acoustic | Log-Pitch | Stdv. |
| Acoustic | 1st Formant | Mean |
| Acoustic | 2nd Formant | Mean |
| Acoustic | 3rd Formant | Mean |
| Acoustic | Harmonicity | Mean |
| Acoustic | Prosodic Jitter | Median |
| Acoustic | Prosodic Shimmer | Median |
| Acoustic | Speech Pausing Time | % of time |
| Acoustic | Vowel Space Area | n/a |
